# 

**Published:** 1840-07

**Authors:** 


### CONTENTS

OF NO. VII.

### ORIGINAL COMMUNICATIONS.

### ESSAYS AND CASES.

Art. I.—Remarks on the Medical Statistics of Natchez—a comparison of its mortality while medicine was protected, and since the introduction of the "Reformed Practice." By Samuel A. Cartwright, M.D. - - 1

Art. II.—Practical Observations on the use of Sulphate of Quinine, By John W. Monette, M. D. - - 21

Art. III.—Report of a Case in which a Testis was found, post mortem, in the Abdomen of a Man. By George W. Bayless, M. D. - - - - - - - - 32

Art. IV.—A Case of Paralysis of the Bladder terminating fatally. By F. Walton Todd, M. D. - - - 34

### REVIEWS.

Art. V.—Crania Americana; or a comparative view of the Skulls of various Aboriginal nations of North and South America; to which is prefixed an Essay on the

Varieties of the Human Species. Illustrated by 78 plates, and a colored map. By SAMUEL GEO. MORTON, M. D., Professor of Anatomy in the Medical Department of Pennsylvania College, &c. - - 35

SELECTIONS FROM AMERICAN AND FOREIGN JOURNALS.

Case of Introsusceptio cured by forcing Air into the Intestines. 57
Dividing the Internal Rectus Muscle for the cure of Squinting 58
The Prevention of Tnrbercles. - - - - - - - 60
The Adulteration of Sulphate of Quinine. - - - - 60
A new Monstrosity. - - - - - - - - - 61
Facts confirmatory of the Value of Vaccination. - - 65
Oil of Codfish in Scrofulous Diseases. - - - - - 67
Treatment of Syphilis - - - - - - - - - 67
New Treatment of Cancer. - - - - - - - - 68

ORIGINAL INTELLIGENCE.

Medicine in Paris, by Dr. LINTON. - - - - - - 69
The Natchez Tornado. - - - - - - - - - 74
Sick Headache, Lobelia in - - - - - - - - 78
Saturated Alcoholic Tincture of Eupatorium Perfoliatum. - 79
Congenital Fungus Hæmatodes. - - - - - - - 79
Milk-Sickness. - - - - - - - - - - 80
Case of Partial Paralysis in Children. - - - - - - 82
Medical Miscellany. - - - - - - - - - 84

